# Atraumatic splenic rupture in a patient treated with apixaban: A case report

**DOI:** 10.1016/j.ijscr.2020.04.050

**Published:** 2020-05-11

**Authors:** Ho-Cing Victor Yau, Sharin Pradhan, Lingjun Mou

**Affiliations:** a Sir Charles Gairdner Hospital, General Surgery, WA, Australia; b General Surgery, Level 8 Sir Charles Gairdner Hospital, Hospital Avenue, Nedlands, WA, 6009, Australia; c Sir Charles Gairdner Hospital, General Surgery – Western Australia Liver and Kidney (WALK), WA, Australia

**Keywords:** Atraumatic splenic rupture, Apixiban, Splenectomy

## Abstract

**Introduction:**

Splenic rupture is often seen in the context of significant trauma. Atraumatic ruptures are described in the context of malignancy, inflammation or infection directly affecting the spleen. Splenic ruptures occurring in patients taking apixaban, a factor Xa inhibitor, are challenging due to the scarcity of a direct reversal agent.

**Presentation of case:**

A 66 year old male presented with syncope and back pain and was found to be haemodynamically unstable and tender in the left upper quadrant. There was no preceding trauma. He has a background of extensive major arterial reconstruction and was taking apixaban for atrial fibrillation. A diagnosis of splenic rupture was confirmed on cross sectional imaging. A laparotomy and splenectomy was necessitated as salvage therapy following splenic artery embolisation. He recovered well and was discharged home 6 days after laparotomy.

**Conclusion:**

Definitive intervention is required for the management of splenic ruptures. A high level of clinical suspicion should be maintained in patients taking direct oral anticoagulants presenting with haemodynamic instability. Open splenectomy remains the most definitive option for treatment of atraumatic rupture in anticoagulated patients.

## Highlights

• Atraumatic splenic rupture should be suspected in unstable patients on direct oral anticoagulants.
• Splenic artery embolisation is an option for definitive therapy.
• Splenectomy can be utilised as salvage therapy.

## 1 Introduction

Splenic rupture is typically described as a result of trauma. Atraumatic ruptures can occur as a result of infection, malignancy, inflammation and as complications of various procedures such as colonoscopy [1]. Splenic rupture in patients on direct oral anticoagulants (DOACs) present unique problems in managing initial resuscitation and optimising patient coagulation due to scarcely available reversal agents. We describe the case of an atraumatic splenic rupture in a patient taking apixaban, a DOAC without an available reversal agent. This case has been reported in accordance with the SCARE criteria [2].

## 2 Presentation of case

A 66 year old male was transferred to the emergency department after an episode of syncope on a cruise ship. This was preceded by sudden onset tearing upper back pain. He had recently recovered from vomiting and diarrhoea secondary to a norovirus infection. He was peripherally pale and clammy with a heart rate of 105 beats per minute, a blood pressure of 66/40 mmHg, respiratory rate of 20, oxygen saturations of 99% on room air and afebrile. He was tender in the right upper quadrant.

He had previously had a type A and type B aortic dissection nine years ago that required an aortic, innominate artery and left common carotid artery graft. Due to further issues with aortic dissection and aneurysm, a thoracic endovascular aortic repair was performed and extended down to his left common iliac and right femoral artery. Grafts for his celiac, superior mesenteric and renal arteries were also present. His other medical history included atrial fibrillation and post cardiac surgery embolic strokes for which he had no residual neurological deficits. His medications were apixaban 5 mg twice a day, amlodipine 10 mg once a day, telmisartan 80 mg once a day, aspirin 100 mg once a day, atorvastatin 20 mg once a day, metoprolol 100 mg at night and 50 mg in the morning and citalopram 20 mg once a day.

**Fig. 1:**
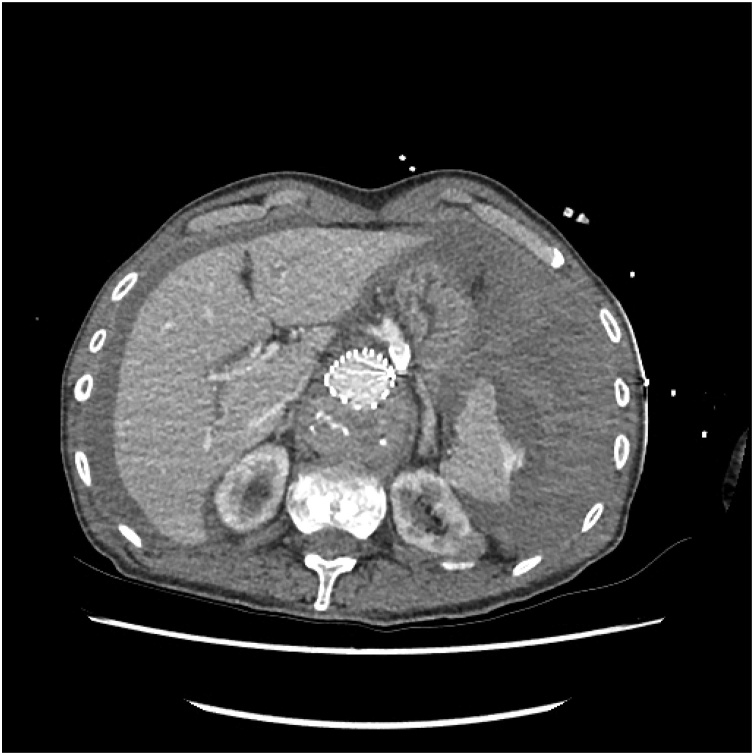
Splenic rupture with haemoperitoneum, contrast blush at the splenic capsule and previous endovascular intervention.

**Fig. 2:**
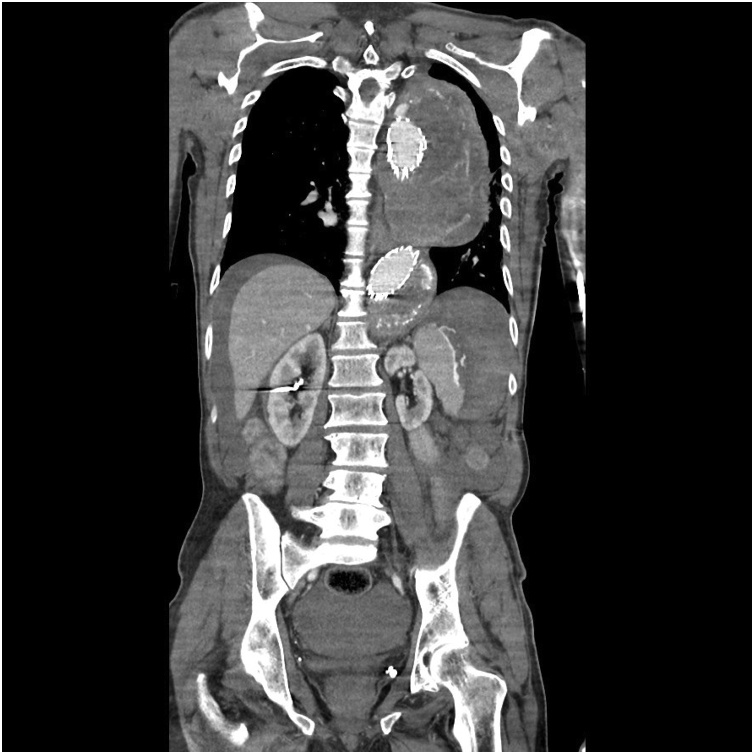
Sagittal image of computed tomography angiogram demonstrating splenic rupture, haemoperitoneum and remnant arterial aneurysm sac.

His initial blood tests showed a haemoglobin of 64 g/L, platelet count of 127 × 10^9^/L, a respiratory alkalosis, lactate of 2.4 mmol/L and creatinine of 100 umol/L. His International Normalised Ratio was 1.8, activated partial thromboplastin time was normal at 38.1 s and fibrinogen was 1.8 g/L. A computed tomography angiogram demonstrated an active splenic haemorrhage with contrast extravasation on the lateral aspect of the spleen associated with capsular stripping (Fig. 1, Fig. 2). There was a large volume haemoperitoneum. The fenestrated aortoiliac graft was patent as were its coeliac, superior mesenteric artery and bilateral renal artery stents. The inferior mesenteric artery origin was thrombosed. Type II endoleaks were seen at the level of the aortic arch and at the level of the L2 lumbar arteries.

**Fig. 3:**
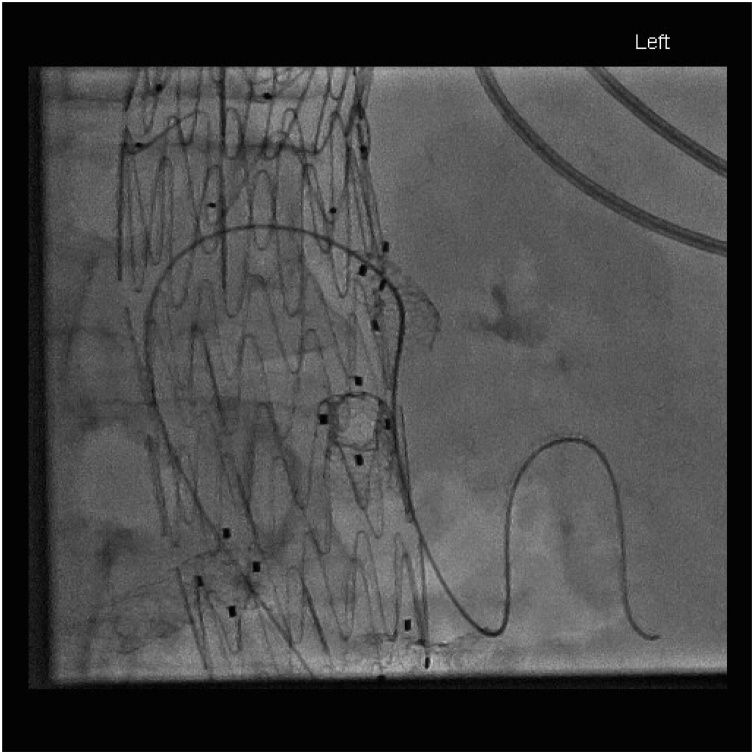
Fluoroscopic images demonstrating wiring of splenic artery.

**Fig. 4:**
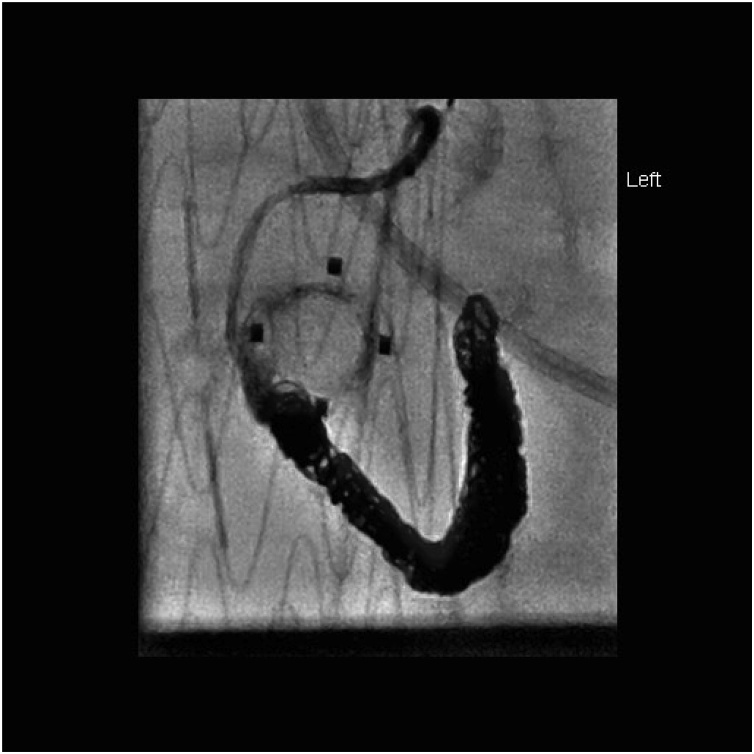
Post embolisation fluoroscopy of splenic artery.

**Fig. 5:**
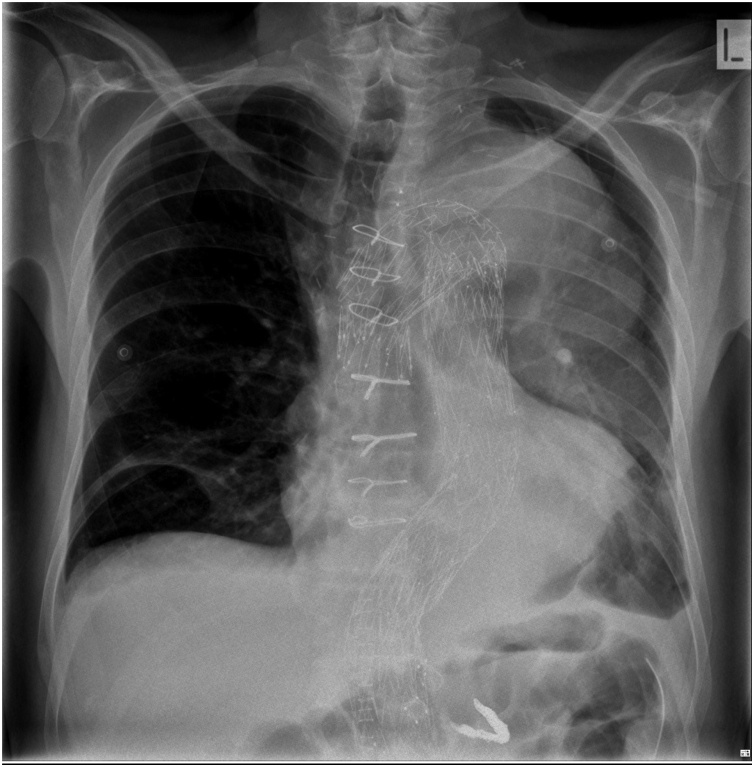
Chest x-ray post operatively demonstrating previous sternotomy wires, endovascular grafts, embolisation coils within the splenic artery and intra-abdominal drain.

After initial resuscitation, blood product replacement (6 units of packed red cells, 10 units of cryoprecipitate, 2 units of Fresh frozen plasma and one unit of platelets) and administration of prothrombin complex concentrate, he was taken for splenic artery embolisation. Extensive coil embolisation was performed with good effect (Fig. 3, Fig. 4, Fig. 5). A small amount of collateral filling of the spleen was apparent via the dorsal pancreatic artery. His post embolisation recovery was in the High Dependency Unit where he stayed haemodynamically stable but was transfusion dependent. He was taken for emergency laparotomy and splenectomy. A laparoscopic approach was not considered due to the expected volume of haematoma in the abdomen and concerns about ongoing bleeding. 2.4 L of blood and haematoma was found as well as a lacerated and shattered spleen. The splenic artery was isolated and ligated, the gastrosplenic and splenocolic ligaments were divided and a vascular stapler was used across the splenic hilum.

His recovery was uneventful and his post splenectomy vaccinations were given on the day of discharge, 6 days after his splenectomy. He was commenced on antibiotic prophylaxis as per local protocol [3]. His stool was positive for norovirus though his nausea, diarrhoea and vomiting had resolved prior to presentation. Histology was consistent with a ruptured spleen with anterior and posterior areas of capsular rupture. Microvascular examination was consistent with rupture as there were occasional vessels containing fibrin and neutrophilic infiltrate adjacent to area of haemorrhage. There was no evidence of malignancy.

## 3 Discussion

Splenic ruptures are often seen in the context of trauma. Atraumatic ruptures are usually reported to be due to malignancies, viral infectious disorder and local inflammatory disorders [1]. A meta-analysis of atraumatic splenic ruptures reported that men were at greater risk of atraumatic rupture and that the mortality rate was 12.2% [1].

Four criteria for spontaneous splenic rupture by Orloff and Peskin and a 5th proposed by Crate and Payne are often referred to in the literature. These include no history of trauma or unusual effort that could injure the spleen, no evidence of disease in other organs that would affect the spleen, no evidence of perisplenic adhesion/scarring to suggest prior trauma or recent rupture, normal microscopic and macroscopic examination of the spleen and no serological evidence of recent viral infections known to be associated with splenic involvement [4,5].

Splenic rupture has been reported to occur in patients administered anticoagulation therapies including heparin, heparin derivatives, tissue plasminogen activator and DOACs [[6], [7], [8], [9], [10], [11], [12], [13], [14], [15], [16], [17]]. It has been proposed that rupture is the result of these medications exacerbating the effects of microtrauma [18].

There is a growing number of reports of atraumatic ruptures in patients taking DOACs [[10], [11], [12], [13], [14], [15], [16]]. These drugs are indicated in the management of atrial fibrillation, pulmonary embolism, deep vein thrombosis and thromboprophylaxis. They present a challenge in establishing normal coagulation function due to difficulties in reversing their anticoagulant actions. Idarucizumab has been developed for reversal of dabigatran, a factor II antagonist, and has limited availability [19]. Apixaban thus far has no available reversal agent in Australia though Andexanet Alfa has been developed as a general reversal agent for rivaroxaban and apixaban [20]. Current recommendations for managing coagulation include administering activated charcoal if the last dose was within 2 hours, the use of prothrombin complex concentrates and recombinant activated factor VII [21,22].

Diagnosis of splenic rupture should be suspected in patients with sudden left upper quadrant pain who are haemodynamically unstable or syncopal. Ultrasound would demonstrate free fluid in the abdomen. Confirmation of the diagnosis is based on the presence of contrast extravasation in a spleen with perisplenic haematoma on cross sectional intravenous contrast studies. Management following initial resuscitation and attempted reversal of any anticoagulants is either splenic artery embolisation or splenectomy. Laparotomy and splenectomy is a salvage option following embolisation. Of the eight case reports of atraumatic splenic rupture in patients on DOACs, five underwent initial therapy with embolisation and three were immediately taken to surgery [[10], [11], [12], [13], [14], [15], [16], [17]]. Three of the five patients who underwent initially embolisation proceeded to laparotomy and splenectomy for ongoing bleeding and transfusion requirements.

This case of atraumatic splenic rupture in a patient taking a DOAC is the first in a patient with extensive endovascular stenting and repair for previous dissection. Whilst he reported no history of significant trauma prior to his presentation, he may have experienced microtrauma as a result of his recent norovirus symptoms. Repeated vomiting may have placed strain on the splenophrenic ligament. We hypothesise that the failure of splenic artery embolisation may have been due to significant collateral arterial supply to the spleen as result of chronic macrovascular disease.

## 4 Conclusion

Resuscitation of patients with life threatening bleeding whilst taking a DOAC remains challenging and a high degree of suspicion for splenic rupture in patients without trauma must be maintained. Potential reversal agents for the various DOACs may improve the initial resuscitative efforts however definitive intervention is still required. Careful monitoring to assess if salvage therapy is required remains essential.

## Declaration of Competing Interest

None.

## Sources of funding

There were no sources of funding.

## Ethical approval

This case report is exempt from ethical approval.

## Consent

The patient for this case report has given his consent for his case and any images to be published in a de-identified manner. Written informed consent was obtained from the patient and this can be provided to the Editor in Chief on request.

## Author contribution

All authors were involved in the care of the patient.

HO-CING VICTOR YAU: Manuscript writing, Critical review of literature.

SHARIN PRADHAN: Primary operator for surgery, Manuscript proof reading,

LINGJUN MOU: Consultant in charge of case, assistant for surgery.

## Registration of research studies

NA.

## Guarantor

Ho-Cing Victor Yau (Primary author) is the guarantor for this case report.

## Provenance and peer review

Not commissioned, externally peer-reviewed.

## References

[1] Renzulli P., Hostettler A., Schoepfer A.M., Gloor B., Candinas D. (2009). Systematic review of atraumatic splenic rupture. Br. J. Surg..

[2] Agha R.A., Borrelli M.R., Farwana R., Koshy K., Fowler A.J., Orgill D.P. (2018). The SCARE 2018 statement: updating consensus Surgical CAse REport (SCARE) guidelines. Int. J. Surg..

[3] Kanhutu K., Jones P., Cheng A.C., Grannell L., Best E., Spelman D. (2017). Spleen Australia guidelines for the prevention of sepsis in patients with asplenia and hyposplenism in Australia and New Zealand. Intern. Med. J..

[4] Orloff M.J., Peskin G.W. (1958). Spontaneous rupture of the normal spleen; a surgical enigma. Int. Abstr. Surg..

[5] Crate I.D., Payne M.J. (1991). Is the diagnosis of spontaneous rupture of a normal spleen valid?. J. R. Army Med. Corps.

[6] Burg M.D., Dallara J.J. (2001). Rupture of a previously normal spleen in association with enoxaparin: an unusual cause of shock. J. Emerg. Med..

[7] Weiss S.J., Smith T., Laurin E., Wisner D.H. (2000). Spontaneous splenic rupture due to subcutaneous heparin therapy. J. Emerg. Med..

[8] Cheung P.K., Arnold J.M., McLarty T.D. (1990). Splenic hemorrhage: a complication of tissue plasminogen activator treatment. Can. J. Cardiol..

[9] Gardner-Medwin J., Sayer J., Mahida Y.R., Spiller R.C. (1989). Spontaneous rupture of spleen following streptokinase therapy. Lancet (London, England).

[10] Amin A., Safaya A., Ronny F., Islam H., Bhuta K., Rajdeo H. (2016). Hemorrhagic shock from spontaneous splenic rupture requiring open splenectomy in a patient taking rivaroxaban. Am. Surg..

[11] Basnet S., Mohanty E., Mir I., Dhital R., Koirala A., Tachamo N. (2019). Atraumatic splenic rupture associated with apixaban. SAGE Open Med. Case Rep..

[12] Gonzva J., Patricelli R., Lignac D. (2014). Spontaneus splenic rupture in a patient treated with rivaroxaban. Am. J. Emerg. Med..

[13] Lowry L.E., Goldner J.A. (2016). Spontaneous splenic rupture associated with apixaban: a case report. J. Med. Case Rep..

[14] Nagaraja V., Cranney G., Kushwaha V. (2018). Spontaneous splenic rupture due to rivaroxaban. BMJ Case Rep..

[15] Naseem Z., Mustaev M., Strekozov B. (2016). Spontaneous splenic rupture secondary to rivaroxaban: rare but raising. Int. J. Surg. Med..

[16] Hattab Y.A., Speredelozzi D., Bajwa O. (2015). Rivaroxaban Causing Spontanuos Splenic Rupture.

[17] Charles Haviland M., Jonathan S., Keith B., James S. (2012). Spontaneous splenic hemorrhage after initiation of dabigatran (Pradaxa) for atrial fibrillation. Am. J. Emerg. Med..

[18] Debnath D., Valerio D. (2002). Atraumatic rupture of the spleen in adults. J. R. Coll. Surg. Edinb..

[19] Pollack C.V., Reilly P.A., van Ryn J., Eikelboom J.W., Glund S., Bernstein R.A. (2017). Idarucizumab for dabigatran reversal — full cohort analysis. N. Engl. J. Med..

[20] Connolly S.J., Crowther M., Eikelboom J.W., Gibson C.M., Curnutte J.T., Lawrence J.H. (2019). Full study report of andexanet alfa for bleeding associated with factor Xa inhibitors. N. Engl. J. Med..

[21] Enriquez A., Lip G.Y., Baranchuk A. (2016). Anticoagulation reversal in the era of the non-vitamin K oral anticoagulants. Europace: Eur. Pacing Arrhythmias Cardiac Electrophysiol..

[22] Suryanarayan D., Schulman S. (2014). Potential antidotes for reversal of old and new oral anticoagulants. Thromb. Res..

